# HOXA10 promotion of HDAC1 underpins the development of lung adenocarcinoma through the DNMT1-KLF4 axis

**DOI:** 10.1186/s13046-021-01867-0

**Published:** 2021-02-17

**Authors:** Tiangang Ma, Bingdi Yan, Yanbing Hu, Qinghua Zhang

**Affiliations:** 1 Department of Respiratory and Critical Care Medicine, the 2nd Hospital of Jilin University, No. 218, Ziqiang Street, Nanguan District, Changchun, 130041 P.R. China; 2 Department of Ultrasound, the 2nd Hospital of Jilin University, Changchun, 130041 P.R. China

**Keywords:** HOXA10, HDAC1, DNMT1, KLF4, Lung adenocarcinoma, Deacetylation, Methylation

## Abstract

**Background:**

Previous research has highlighted the ability of Homeobox A10 (HOXA10) to the promote proliferation, migration, and epithelial-mesenchymal transformation of various cancers, including lung adenocarcinoma (LAD), which is characterized by an aggressive disease course that exhibits rapid proliferation and migration, with studies suggesting histone deacetylase 1 (HDAC1) to be a downstream mediator of HOXA10. The current study aimed to investigate the mechanism by which HOXA10-mediated HDAC1 influences the development of LAD.

**Methods:**

The expression patterns of HOXA10, HDAC1, DNA methyltransferase 1 (DNMT1), and Kruppel-like factor 4 (KLF4) were determined. Additionally, the effect of HOXA10, HDAC1, or DNMT1 on invasive phenotypes of LAD was analyzed using depletion experiments. The interactions among HOXA10, HDAC1, DNMT1, and KLF4 were evaluated via chromatin immunoprecipitation, dual luciferase assay or co-immunoprecipitation. Furthermore, the tumorigenic ability of the LAD cells following HOXA10 silencing and/or HDAC1 overexpression in vivo was also investigated.

**Results:**

In the LAD tissues and cells, HOXA10, HDAC1, and DNMT1 all exhibited high levels of expression, while KLF4 was poorly expressed. HOXA10 silencing inhibited the expression of HDAC1, reduced LAD cell proliferation, migration, and invasion, and promoted the apoptosis. HDAC1 promoted DNMT1 expression through deacetylation, and DNMT1 inhibited the KLF4 expression through DNA methyltransferase. The in vitro findings were further attested through the use of in vivo assays.

**Conclusion:**

Taken together, the key observations of the current study highlight the role of HOXA10 and HDAC1 in promoting the proliferation and migration of LAD cells. HOXA10-induced upregulation of HDAC1 interacts with DNMT1-KLF4 axis, while the inhibition of HOXA10 or HDAC1 represents a promising anti-tumor therapy target for LAD.

**Supplementary Information:**

The online version contains supplementary material available at 10.1186/s13046-021-01867-0.

## Background

Lung cancer morbidity and mortality continue to exhibit progressive increases year by year, with studies highlighting it as the most common malignant tumor worldwide of which lung adenocarcinoma (LAD) represents the most common histological subtype of lung cancer [1]. Tobacco consumption is the main cause of lung cancer, and in addition, genetic factors, diet, occupational exposure, as well as air pollution have all been implicated in the epidemiology of lung cancer independently or in concert with smoking [2]. Although some progress has been made in the arena of lung cancer therapeutics, the overall survival is still relatively low owing to earlier chemotherapy resistance and late stage diagnosis [3]. A greater understanding of the finer molecular mechanism underlying LAD cell proliferation and metastasis is crucial in order to prevent tumor metastasis and improve patient survival.

As a member of the HOX family, Homeobox A10 (HOXA10) has been shown to play a notable role in the regulation of various cellular functions in various kinds of diseases and cancers, including gastric cancer, nasopharyngeal carcinoma, and prostate carcinoma [4–6]. Moreover, HOXA10 has been demonstrated to promote the occurrence of LAD [7]. Histone deacetylase 1 (HDAC1) represents a deacetylase that has been implicated in the occurrence and development of various cancer in addition to exerting potential functions on cell functions [8]. A relationship between HDAC1 and lung cancer has been previously emphasized, with HDAC1 suggested as a functional diagnostic and prognostic indicator of lung cancer [9]. Functionally speaking, HOXA10 has been identified as a stimulatory factor for the transcription of HDAC1 [10]. Du Z et al., demonstrated that HDAC1 can stabilize DNA methyltransferase 1 (DNMT1), the primary enzyme maintaining DNA methylation [11]. Moreover, via methylation means, DNMT1 has been shown to inhibit the expression of Krüppel-like factor 4 (KLF4) [12], a member of KLF transcription factor family, which inhibited the invasion and metastasis of LAD cells [13]. Based on the aforementioned exploration of evidence, we put forward the hypothesis that HOXA10 promotes the development of LAD through downregulating KLF4 mediated by histone deacetylase HDAC1 and DNMT1. In order to prove our hypothesis, in vitro assays as well as in vivo experiments were performed to elucidate the interactions among HOXA10, HDAC1, DNMT1, and KLF4 in LAD in an attempt to identify the detailed potential mechanism underlying the treatment of LAD.

## Methods

### Clinical sample collection

From October 2017 to December 2018, 42 cases of LAD and paracancerous tissues (> 3 cm away from tumor tissues) were collected from patients diagnosed with LAD (25 males and 17 females), all of whom were pathologically confirmed and surgically treated at the Second Hospital of Jilin University. Of the 42 cases, 29 cases were under 55 years and 13 cases were over 55 years. The tumor tissues were either classed as well differentiated (*n* = 20), moderately differentiated (*n* = 12), or poorly differentiated (*n* = 10). Moreover, lymph node metastasis was detected in 16 cases while no such findings were identified in the remaining 26 cases. According to latest staging system for lung cancer published by the International Union Against Cancer and American Joint Committee on Cancer [14], the enrolled cases were further divided into stage II (*n* = 27) and stage IIIa (*n* = 15). Patients who received any medication, radiochemotherapy or immunobiological therapy were excluded from the study.

### Cell culture

LAD cell lines GLC-82 (ZY-H065), XWLC05 (HTX-2446), and SPCA-1 (BSC-5307481030-01) were purchased from the American Type Culture Collection (Manassas, VA, USA), H1299 (3111C0001CCC000469) and A549 (3111C0001CCC000002) with the human bronchial epithelial (HBE) cells (3111C0001CCC000174) purchased from the Cell Resource Center of the Institute of Basic Medical Sciences, Chinese Academy of Medical Sciences (Beijing, China). GLC-82, SPCA-1, and HBE cells were cultured in Dulbecco’s modified Eagle’s medium (GIBCO-BRL, CA, USA) containing 10% fetal bovine serum (FBS) (GIBCO-BRL), H1299 and XWLC05 cells were cultured in Roswell Park Memorial Institute Medium 1640 medium (Shanghai Yuanmu Biotechnology Co., Ltd., Shanghai, China) containing 10% FBS, and A549 cells were cultured in McCoy’s 5A Media (Modified with Tricine) containing 10% FBS.

### Cell transfection

Three short hairpin RNAs (shRNAs) targeting HOXA10, HDAC1, DNMT1, and KLF4 in addition to one scramble shRNA against negative control (sh-NC) were cloned into pLKO.1-puro (Addgene, Cambridge, MA, USA), respectively. The shRNAs and lentiviral packaging vectors were co-transfected into HEK293T cells using Lipofectamine 2000 reagent (Invitrogen, Carlsbad, CA, USA) as per the manufacturer’s protocol for 48–72 h, after which the supernatant was collected to infect target cells at later stage. The coding sequences of HDAC1 and DNMT1 were amplified, digested with *HindIII*/*EcoRI* (Thermo Fisher Scientific, Waltham, MA, USA), and inserted into *HindIII*/*EcoRI*-digested pcDNA3.1 (+) (Addgene). HOXA10-pcDNA3.1 was transfected into the LAD cells with the overexpression effect subsequently confirmed by Western blot analysis 48 h post transfection. The shRNA sequences are depicted in Table 1.

**Table 1:** Interference sequences for cell transfection

Gene	Sequence
HOXA10	shRNA-1: 5′-CTTTCGCGCAGAACATCAA-3′
	shRNA-2: 5′-TATGTACCTTACTCGAGAG-3′
	shRNA-3: 5′-TGAATCGAGAAAACCGGAT-3′
HDAC1	shRNA-1: 5′-AGCGACGACTACATCAAATTC-3′
	shRNA-2: 5′-ATGGCTATACCATCCATAATG-3′
	shRNA-3: 5′-AGACCCTGACAAACCAATTTC-3′
DNMT1	shRNA-1: 5′-CCAUGAGCACCGUUCUCCTT-3′
	shRNA-2: 5′-GGAGAACGGUGCUCAUGGTT-3′
	shRNA-3: 5′-TTGATGTCAGTCTCATTGG-3′
KLF4	shRNA-1: 5′-TACCCATCCTTCCTGCCCGAT-3′
	shRNA-2: 5′-ATCGGTCATCAGCGTCAGCAA-3′
	shRNA-3: 5′-AAGTCATCTTGTGAGTGGATAA-3′
scrambled shRNA	5′-GCGATGGGCGAACTGACACG-3′

Note: *HOXA10* Homeobox A10, *HDAC1* Histone deacetylase 1, *DNMT1* DNA methyltransferase 1, *KLF4* Kruppel-like factor 4, *shRNA* Short hairpin RNA

### Reverse transcription quantitative polymerase chain reaction (RT-qPCR)

Total RNA was extracted from the tissues using TRIzol (Invitrogen, Carlsbad, CA, USA), after which cDNA was obtained through reverse transcription using a reverse transcription kit (RR047A, Takara, Japan). RT-qPCR was performed using a SYBR® Premix Ex TaqTM II (Perfect Real Time) kit (DRR081, Takara, Japan) in a real-time PCR instrument ABI 7500 (ABI, Foster City, CA, USA). Primers were synthesized by Shanghai Biotech Co., Ltd. (Shanghai, China) (Table 2). The Ct value of each well was recorded, with glyceraldehyde-3-phosphate dehydrogenase (GAPDH) employed as the internal reference. The 2^-ΔΔCt^ formula was applied to calculate relative expression.

**Table 2:** Primer sequences for RT-qPCR

Gene	Primer sequence
HOXA10	F: 5′-AGAGATTAGCCGCAGCGTCC-3′
	R: 5′-TTCCTGGGCAGAGCCTGAAG-3’
HDAC1	F: 5′-CTACTACGACGGGGATGTTGG-3′
	R: 5′-GAGTCATGCGGATTCGGTGAG-3′
DNMT1	F: 5′-AACCTTCACCTAGCCCCAG-3′
	R: 5′-TGACAGGTGGTCACTCCTCATG-3′
KLF4	F: 5′-TTCTCCACGTTCGCGTCCGG-3′
	R: 5′-TCTCGCCAACGGTTAGTCGGGG-3′
GAPDH	F: 5′-CACCCACTCCTCCACCTTTG-3′
	R: 5′-CCACCACCCTGTTGCTGTAG-3′

Note: *HOXA10* Homeobox A10, *HDAC1* Histone deacetylase 1, *DNMT1* DNA methyltransferase 1, *KLF4* Kruppel-like factor 4, *GAPDH* Glyceraldehyde-3-phosphate dehydrogenase, *F* Forward, *R* Reverse, *RT-qPCR* Reverse transcription quantitative polymerase chain reaction

### Western blot analysis

The LAD cells and tissues were lysed using radioimmunoprecipitation assay lysis buffer (Beijing Solarbio Science & Technology Co., Ltd., Beijing, China), after which the protein concentration was measured using a bicinchoninic acid protein assay kit (Thermo Fisher Scientific). The protein was subsequently separated using sodium dodecyl sulfate-polyacrylamide gel electrophoresis (SDS-PAGE), and transferred to a nitrocellulose membrane (Millipore, Bedford, MA, USA) which was blocked, and incubated with the primary antibodies overnight. Protein bands were detected using an enhanced chemiluminescence detection kit (Pierce Biotechnology, Rockford, IL, USA). The used primary antibodies were anti-HOXA10 (ab23392, 1: 500), anti-HDAC1 (ab7028, 1: 2000), anti-DNMT1 (ab134148, 1: 1000), anti-KLF4 (ab215036, 1: 1000), anti-HA (ab130275, 1: 1000), anti-GAPDH (ab181602, 1: 10000), and the secondary antibody was IgG (ab6721, 1: 2000). All mentioned antibodies were obtained from Abcam (Cambridge, MA, USA). Image J software (http://rsb.info.nih.gov/ij/, Bethesda, MD, USA) was used to quantify the gray scale of the detected protein.

### Cell counting kit-8 (CCK-8) assay

The transfected LAD cells were seeded into 96-well plates at a density of 3 × 10^3^ cells/well, then, after 0, 24, 48 and 72 h, CCK-8 solution (Signalway Antibody, College Park, MD, USA) was added to each well and incubated for 1 h. The absorbance (optical density value) was measured at a wavelength of 450 nm on a microplate spectrophotometer (Thermo Fisher Scientific).

### Flow cytometry analysis of cell cycle and apoptosis

Cell cycle analysis was performed using cells that were treated with antibody to 5-bromo-2′-deoxyuridine-fluorescein isothiocyanate (FITC) (BD Biosciences, Franklin Lakes, NJ, USA) and DNA was stained with 7-AminoactinomycinD (7-ADD, Sigma-Aldrich Chemical Company, St Louis, MO, USA) [15].

Cell apoptosis analysis was performed in line with an Annexin V-FITC kit (Shanghai Beyotime Biotechnology Co., Ltd., Shanghai, China) which was used for staining, with both the DNA content and apoptosis analyzed using a FACScan flow cytometer (BD Biosciences).

### Scratch test

When the cell confluence reached approximately 90%, sterile pipette tips were used to scratch the middle of the cells. The cells were cultured in serum-free medium for 24 h and evaluated accordingly. ImagePro Plus analysis software 7.0 (Media Cybernetics, Inc., Rockville, MD, USA) was employed to determine the migration distance.

### Transwell assay

The upper chamber of the Transwell chamber was covered with Matrigel (50 mL; 356,234; Becton, Dickinson and Company, NJ, USA). The transfected cells were then seeded into the upper cavity of the Transwell filter membrane and incubated for 48 h in serum-free medium. The infiltrated cells on the underside of the filter membrane were fixed with 5% glutaraldehyde, stained with 0.1% crystal violet and counted under the microscope. The invasion was determined by the number of cells that passed through the Matrigel.

### Dual luciferase reporter gene assay

The binding site on the HDAC1 promoter to HOXA10 and the binding site on the KLF4 promoter to DNMT1 were mutated by PCR. The wild-type (WT) and mutant (MUT) sequences of the HDAC1 or KLF4 promoter were inserted into the pGL3-vector (Promega Corporation, Madison, WI, USA). The luciferase vector pRL-TK (Promega) and pGL3-HDAC1 promoter vector were transfected into the cells in the presence of sh-HOXA10 or sh-NC, respectively. Similarly, the pRL-TK and pGL3-KLF4 promoter vectors were transfected into the cells in the presence of sh-DNMT1 or sh-NC. After 48 h had elapsed, the luciferase activity was determined using a Dual Luciferase Reporter Assay System (Promega) normalized to Renilla luciferase activity. The activity control for co-transfection with the pRL-RK and pGL3-HDAC1 promoters was set as 1.0.

### Chromatin immunoprecipitation (ChIP)

A ChIP kit (Millipore) was used to investigate the enrichment of HOXA10 in the HDAC1 promoter region and the enrichment of DNMT1 in the KLF4 promoter region. Cells at the logarithmic growth phase from group were collected, fixed with formaldehyde and sonicated. The negative control IgG antibodies (ab205718, 1: 50, Abcam) and target protein-specific antibody HOXA10 (ab23392, 1: 500, Abcam) respectively. DNMT1 (ab13537, 1: 50, Abcam) were added for incubation at 4 °C overnight. After that, endogenous DNA-protein complexes were precipitated and de-crosslinked. Finally, DNA fragments were extracted and purified, and used for testing the binding of HOXA10 to HDAC1 promoter and the binding of DNMT1 to KLF4 promoter. The primer sequences are shown in Table 3.

**Table 3:** Primer sequences for ChIP

Gene	Primer sequence
HDAC1	F: 5′-AAAGAAAGGAAACCTGCCCTC-3′
	R: 5′-TGCAGTCACCCAGGATGACTA-3′
KLF4	F: 5′-CCTGACCATGAAAACTGTGAGATA-3′
	R: 5′-GCTGGTCTTGAACTCCTGCGCTCA-3′

Note: *ChIP* Chromatin immunoprecipitation, *HDAC1* Histone deacetylase 1, *KLF4* Kruppel-like factor 4, *F* Forward, *R* Reverse

### Co-immunoprecipitation (co-IP)

Co-IP analysis was performed using Pierce™ Co-IP kit (Thermo Scientific Pierce, 26,149). First, rabbit anti-human HDAC1 antibody (ab150399, 1: 100, Abcam) and goat anti-rabbit IgG (ab136636, 1: 5000, Abcam) were conjugated with AminoLink Plus Coupling Resin and incubated at room temperature for 120 min. The A549 cells were then lysed using IP lysis buffer solution, with the cell lysates harvested, pre-treated with Pierce Control Agarose Resin and added into the antibody-crosslinked resin. After co-IP at 4 °C overnight, the precipitate was eluted and subjected to Western blot analysis.

### In vitro acetylation assay

Glutathione S-transferase-fused DNMT1 was co-transfected into A549 cells with either sh-NC or sh-HDAC1, and 50 μL acetyltransferase assay buffer was incubated in 20 μM acetyl-CoA at 30 °C for 2 h. The reaction mixture was analyzed by SDS-PAGE and further examined through Western blot analysis.

### In vivo ubiquitination (Ub) test

The A549 cells in the presence of sh-NC and sh-HDAC1 were co-transfected with HA-Ub and pCMV5-myc-DNMT1 plasmids for 2 h, and treated with 10 μg/mL MG132 (Sigma-Aldrich). The cell lysate was then immunoprecipitated with anti-myc antibody (ab172, 1: 250, Abcam), with the protein mixture in the precipitate was separated by SDS-PAGE. Ub-DNMT1 was analyzed with antibody to HA (ab130275, 1: 150, Abcam). The cell lysate was then immunoprecipitated with antibody to DNMT1, and the ubiquitination level of DNMT1 protein in each experimental group was detected by Western blot analysis of antibody to HA.

### Pulse-chase

The supernatant was collected through the pre-spin using 40 μL Agarose A/G beads (Upstate Biotechnology, NY, USA), added with the primary antibody, and immunoprecipitated at 4 °C overnight. Protein samples were denatured in Laemmli buffer (4 ×), separated on a 10% polyacrylamide gel and subjected to Western blot analysis as described above.

### Methylation-specific PCR (MSP)

MSP was performed on sodium bisulfate-treated DNA using the EZ DNA Methylated Gold™ Kit (Zymo Research, Irvine, CA, USA) based on the manufacturer’s instructions. As previously mentioned, methylation-specific primers for the KLF4 promoter were designed using the MethPrimer program. The methylation-specific primers and SYBR-Green reaction mixture were applied for PCR amplification of bisulfite-converted genomic DNA. The primers used are illustrated in Table 4.

**Table 4:** Primer sequences for methylation-specific PCR

Gene	Primer sequence
KLF4 (methylated)	F: 5′-CGTAGGGTTTAAATAGGTGATAACG-3′
	R: 5′-AAATAATAAAAACTCGAACACCGAA-3′
KLF4 (nonmethylated)	F: 5′-TGTAGGGTTTAAATAGGTGATAATGA-3′
	R: 5′-AAATAATAAAAACTCAAACACCAAA-3′

Note: *PCR* Polymerase chain reaction, *KLF4* Kruppel-like factor 4, *F* Forward, *R* Reverse

### Xenograft in nude mice

Eighteen six-week-old female athymic BALB/c nude mice (weight: 18–20 g) were purchased from the Experimental Animal Center of Guangdong Province (Guangdong, China), and reared under specific pathogen-free conditions. Nude mice were intraperitoneally injected with LAD cells (1 × 10^6^ cells/200 μL/mouse) transfected with sh-NC and oe-NC, with sh-HOXA10 and oe-NC, in addition to sh-HOXA10 and oe-HDAC1. The longest diameter (L) and shortest diameter (W) of the tumors in the mice were measured every 4 days post injection with the tumor growth plotted and a data curve constructed based on the formula V = L × W^2^ × 0.5. On day 25, the mice were euthanized and their tumors were excised. Six tumor samples from each group were weighed and averaged.

### Immunohistochemistry

LAD tissue sections were dewaxed with xylene and hydrated with alcohol at descending concentrations. Following antigen retrieval, the tissues were incubated with goat polyclonal antibody to HOXA10 (1: 200; ab191470; Abcam) at 4 °C overnight, as well as with biotinylated rabbit anti-goat secondary antibody (ab97100; Abcam) at 37 °C for 30 min. Diaminobenzidine (Bost Biotechnology Co., Ltd., Wuhan, China) was added stained for 1–2 min, while hematoxylin (KeyGEN Biotech Co., Ltd., Nanjing, Jiangsu, China) was counterstained for 1 min. The tissue sections were subsequently dehydrated and fixed using neutral balm. Five fields of view were selected and observed under an optical microscope (200 ×, Nikon, Tokyo, Japan) with 100 cells from each field analyzed. Brown-yellow cells were considered to be positive. Cells with a staining degree greater than 25% were considered to be positive cells with the positive rate calculated using the following formula: positive rate = (the number of positive cells/the number of total cells) × 100%.

### Statistical analysis

SPSS 22.0 statistical software (IBM Corp. Armonk, NY, USA) was applied for statistical analysis. All data were expressed as the mean ± standard deviation. Data between LAD and paracancerous tissues were compared by paired *t*-test, while data between the other two groups of unpaired design were analyzed using unpaired *t*-test. Data comparison among multiple groups was conducted using one-way analysis of variance (ANOVA) and Tukey’s post-test. Cell viability at different time points was compared using two-factor ANOVA, and tumor volume data at different time points were compared using Bonferroni-corrected repeated measures ANOVA. Pearson correlation or Spearman correlation was employed to analyze the correlation between HOXA10 and HDAC1. *p* < 0.05 was considered to be indicative of statistically significant difference.

## Results

### HOXA10 silencing inhibited the reproduction of LAD cells and promoted their apoptosis

**Fig. 1:**
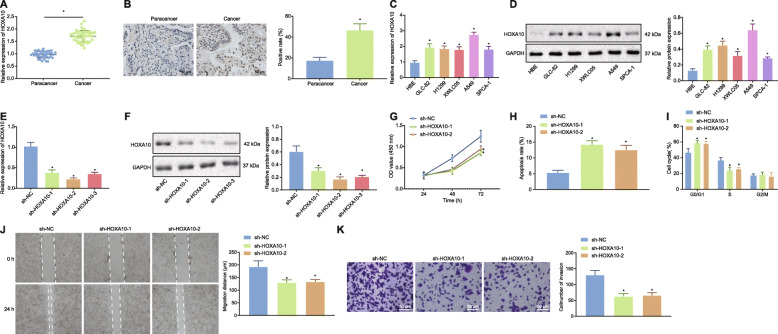
Silencing HOXA10 inhibited LAD cell proliferation and promoted its apoptosis. **a** Expression of HOXA10 in LAD tissues (*n* = 42) and paracancerous tissues (*n* = 42) by RT-qPCR. **b** Immunohistochemical detection of HOXA10 in LAD tissues and paracancerous tissues (200 ×). **c** HOXA10 mRNA expression in HBE cells and 5 LAD cell lines (GLC-82, H1299, XWLC05, A549, and SPCA-1) by RT-qPCR, normalized to GAPDH. **d** HOXA10 protein expression in HBE cells and 5 LAD cell lines by Western blot analysis, normalized to GAPDH. **e** Expression of HOXA10 mRNA in response to different sh-HOXA10 determined by RT-qPCR, normalized to GAPDH. **f** Expression of HOXA10 protein in response to different sh-HOXA10 determined by Western blot analysis, normalized to GAPDH. **g** Cell viability detection by CCK-8. **h** The detection of apoptosis by flow cytometry. I, The cell cycle distribution through flow cytometry. **j** Cell migration by scratch test. **k** Cell invasion by Transwell assay (200 ×). * *p* < 0.05 vs. the paracancerous tissues (panels **a** and **b**), HBE cells (panels **c** and **d**) or A549 cells transfected with sh-NC (panels **e**-**k**). All experiments were done in triplicate

Previous studies have highlighted that HOXA10 is upregulated in LAD tissues and cells [16], a finding that was verified in our study. RT-qPCR was used to detect the expression of HOXA10 in LAD tissues (Fig. 1a), and our data indicated that HOXA10 was highly expressed in LAD tissues. Immunohistochemical detection of HOXA10 in tissues (Fig. 1b) revealed that the protein expression of HOXA10 in LAD tissues was markedly upregulated. In addition, HOXA10 mRNA and protein expression in LAD cell lines (GLC-82, H1299, XWLC05, A549, and SPCA-1) was upregulated compared to HBE cell lines as determined by RT-qPCR and Western blot analysis (Fig. 1c, d). Moreover, the highest expression of HOXA10 was detected in the A549 cells, which were as a result selected for subsequent study and analysis. To explore the effect of HOXA10 on LAD, the sh-HOXA10–2 and sh-HOXA10–3 sequence with the best silencing effect was selected through RT-qPCR and Western blot analysis for next experiments and re-termed as sh-HOXA10–1 and sh-HOXA10–2 (Fig. 1e, f). We subsequently identified cell viability by CCK-8 (Fig. 1g), apoptosis (Fig. 1h) and cycle distribution (Fig. 1i) by flow cytometry, and cell migration and invasion through scratch test and Transwell assay (Fig. 1j, k). The A549 cells transfected with sh-HOXA10–1 and sh-HOXA10–2 exhibited significantly reduced cell viability, significantly increased rate of apoptosis, and a greater number arrested at the G0/G1 phase, while fewer cells were arrested at the S phase, with a significant reduction in relation to migration and invasion. In summary, silencing the expression of HOXA10 can inhibit LAD cell viability, block the cell cycle in the G0/G1 phase, inhibit migration and invasion, and promote the apoptosis of LAD cells.

### HOXA10 promoted the expression of HDAC1 in LAD cells

**Fig. 2:**
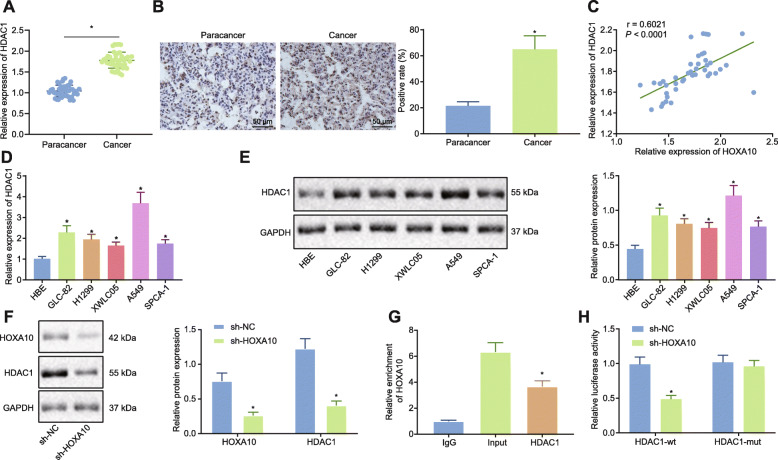
HOXA10 promoted the expression of HDAC1 in LAD cells. **a** Expression of HDAC1 in LAD tissues (*n* = 42) and paracancerous tissues (n = 42) by RT-qPCR. **b** Immunohistochemical detection of HDAC1 in LAD tissues and paracancerous tissues (200×). **c** Pearson correlation of HOXA10 and HDAC1 expression in LAD. **d** HDAC1 mRNA expression in HBE cells and 5 LAD cell lines by RT-qPCR, normalized to GAPDH. **e** HDAC1 protein expression in HBE cells and 5 LAD cell lines by Western blot analysis, normalized to GAPDH. **f** HOXA10 and HDAC1 protein expression in A549 cells after HOXA10 silencing by Western blot analysis normalized to GAPDH. **g** HOXA10 binding to HDAC1 promoter by ChIP. **h** Dual luciferase reporter gene assay to verify the binding between HOXA10 and HDAC1. * *p* < 0.05 vs. the paracancerous tissues (panels **a** and **b**), HBE cells (panels **d** and **e**), A549 cells transfected with sh-NC (panel **f**), DNA-protein complex fragments incubated with IgG (panels **g**), or cells transfected with sh-NC and HDAC1-wt (panel **h**). All experiments were done in triplicate

Previous research has demonstrated that HOXA10 can promote cancer cell proliferation by directly binding to the HDAC1 promoter and upregulating HDAC1 expression in liver cancer cells [10]. In addition, the expression of HDAC1 has been reported to be reversely correlated with the overall survival of patients with lung cancer [9]. However, the regulatory mechanism by which the two interact in LAD is yet to be fully understood. High levels of HDAC1 expression in LAD tissues were detected via RT-qPCR (Fig. 2a). Immunohistochemistry (Fig. 2b) results indicated that HDAC1 protein expression in LAD tissues was significantly upregulated. Pearson correlation analysis (Fig. 2c) revealed a positive correlation between HOXA10 and HDAC1 expression in LAD tissues. Furthermore, RT-qPCR and Western blot analysis were used to detect the expression of HDAC1 in normal cells and LAD cell lines (Fig. 2d, e) while evidence was obtained suggesting that HDAC1 mRNA and protein expressions in LAD cell lines (GLC-82, H1299, XWLC05, A549, and SPCA-1) were significantly upregulated when compared with HBE cell line. Western blot analysis was performed and subsequently demonstrated that the expression of HOXA10 and HDAC1 protein was significantly downregulated after HOXA10 silencing (Fig. 2f). To explore the binding of HOXA10 to the HDAC1 promoter, ChIP was performed (Fig. 2g), and the results revealed that HOXA10 was enriched on the HDAC1 promoter. The dual luciferase reporter gene assay further attested the binding relationship (Fig. 2h). Luciferase activities were significantly decreased in the sh-HOXA10 and HDAC1-WT co-transfection group compared to the sh-NC and HDAC1-WT group, demonstrating that HOXA10 binds to the wild-type HDAC1 promoter; whereas the fluorescence activities were similar between the sh-HOXA10 + HDAC1-MUT co-transfection group and the sh-NC + HDAC1-MUT co-transfection group, validating that the HOXA10 is unable to bind to the mutant HDAC1 promoter. The aforementioned data demonstrated that in LAD cells, HOXA10 promotes HDAC1 expression by binding to its promoter.

### HOXA10 promoted the proliferative, invasive and migrative potentials of LAD cells through HDAC1

**Fig. 3:**
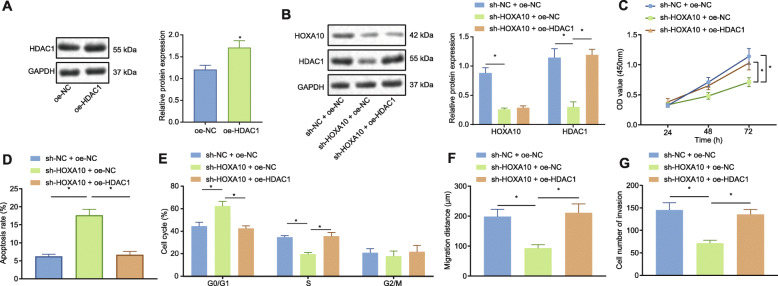
HDAC1 is the mediator of HOXA10 in LAD cells to regulate proliferation, apoptosis, invasion and migration. **a** HDAC1 protein expression following transfection with oe-NC or oe-HDAC1 by Western blot analysis, normalized to GAPDH. A549 cells were transfected with sh-NC + oe-NC, sh-HOXA10 + oe-NC or sh-HOXA10 + oe-HDAC1 for panels B-G. **b** HOXA10 and HDAC1 protein expression by Western blot analysis, normalized to GAPDH. **c** Cell viability detection by CCK-8. **d** The detection of cell apoptosis by flow cytometry. **e** The cell cycle distribution by flow cytometry. **f** Cell migration by scratch test. **g** Cell invasion by Transwell assay. All experiments were done in triplicate. * *p* < 0.05

We subsequently set out to investigate the effects associated with the interaction between HOXA10 and HDAC1 in LAD cells. We initially examined the effects associated with the overexpression and silencing for HOXA10 and HDAC1, which were effective (Fig. 3a, b). The results of CCK-8 experiment (Fig. 3c), flow cytometry (Fig. 3d, e), cell scratch test (Fig. 3f) and Transwell assay (Fig. 3g) illustrated that silencing HOXA10 led to a reduction in cell viability, increased apoptosis rate, increased number of cells at the G0/G1 phase, decreased S-phase-arrested cells, as well as reduced cell migration and invasion, while overexpressing HDAC1 at the same time could reverse the impact of sh-HOXA10 on the above cell functions. Similar tendencies were observed in GLC-82 cells (Supplementary Figure 1). The aforementioned results indicate that silencing HOXA10 inhibits the proliferative, migrative and invasive abilities of LAD cells by inhibiting the expression of HDAC1.

### HDAC1 inhibited the degradation of DNMT1 by promoting its deacetylation

**Fig. 4:**
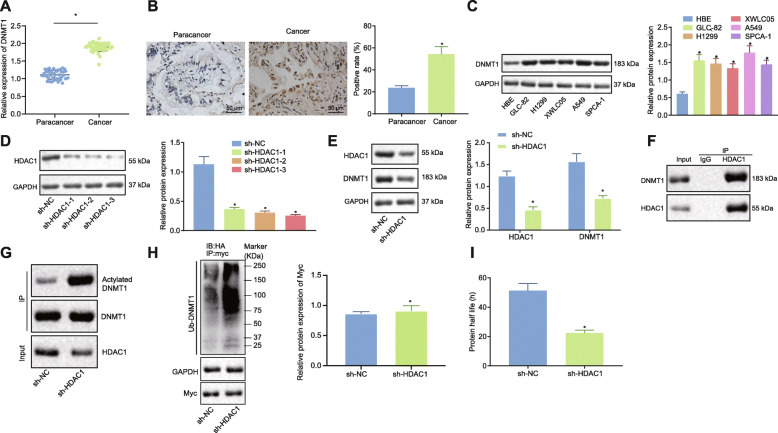
HDAC1 promoted DNMT1 deacetylation. **a** Expression of DNMT1 in LAD tissues (*n* = 42) and paracancerous tissues (*n* = 42) by RT-qPCR, normalized to GAPDH. **b** Immunohistochemical detection of DNMT1 in LAD tissues (*n* = 42) and paracancerous tissues (*n* = 42) (200 ×). **c** DNMT1 expression in HBE cells and 5 LAD cell lines by Western blot analysis, normalized to GAPDH. **d** Silencing efficiency of HDAC1 by Western blot analysis, normalized to GAPDH. **e** The expression of HDAC1 and DNMT1 when HDAC1 was silenced by Western blot analysis, normalized to GAPDH. **f** Co-IP analysis of the interaction between HDAC1 and DNMT1 relative to IgG. **g** In vitro acetylation of DNMT1. **h** In vivo ubiquitination of DNMT1 relative to GAPDH. **i** Pulse tracking analysis for the effect of HDAC1 on the stability of DNMT1. All experiments were done in triplicate. * *p* < 0.05 vs. the paracancerous tissues (panels **a** and **b**), HBE cells (panel **c**) or cell treated with sh-NC (panels **d**, **e**, and **i**)

Previous studies have suggested that HDAC1 can stabilize DNMT1 and promote its expression through its deacetylase function [11]. In our study, RT-qPCR (Fig. 4a) and immunohistochemistry (Fig. 4b) results demonstrated that DNMT1 protein and mRNA were highly expressed in LAD tissues compared to paracancerous tissues. Western blot analysis (Fig. 4c) revealed that the expression of DNMT1 protein in LAD cell lines was significantly upregulated. Then, sh-HDAC1–3 with better silencing effect was selected for follow-up experiments (Fig. 4d). We identified that the protein levels of DNMT1 were significantly downregulated after silencing of HDAC1 (Fig. 4e). The Co-IP (Fig. 4f) results revealed that HDAC1 could bind to DNMT1 in LAD cell lines. In addition, during in vitro acetylation analysis, GST-fused DNMT1 was co-transfected with sh-NC or sh-HDAC1 into A549 cells, followed by incubation with acetyl coenzyme A, the results of which indicated that the degree of DNMT1 acetylation was notably increased in the presence of sh-HDAC1, indicating that HDAC1 inhibited the degree of DNMT1 acetylation (Fig. 4g). As for in vivo ubiquitination determination, A549 cells following transfection using sh-NC and sh-HDAC1 were co-transfected with HA-Ub and pCMV5-myc-DNMT1 plasmids, followed by immunoprecipitation using anti-myc in cell lysates. The obtained protein mixture was then separated by SDS-PAGE and Ub-DNMT1 was analyzed using anti-HA (Fig. 4h). The results obtained indicated that the degree of DNMT1 ubiquitination after sh-HDAC1 treatment was significantly increased. Moreover, pulse tracking experiments further showed at 0, 6, 12, 24 and 48 h after co-transfection of GST-fused DNMT1 with sh-NC and sh-HDAC1, the degradation of DNMT1 in response to sh-HDAC1 was increased, and the half-life was significantly shortened (Fig. 4i). The above results demonstrate that HDAC1 could directly bind to DNMT1, acetylate it and stabilize its expression.

### HDAC1 silencing inhibited the proliferative, migrative and invasive capabilities of LAD cells by downregulating the expression of DNMT1

**Fig. 5:**
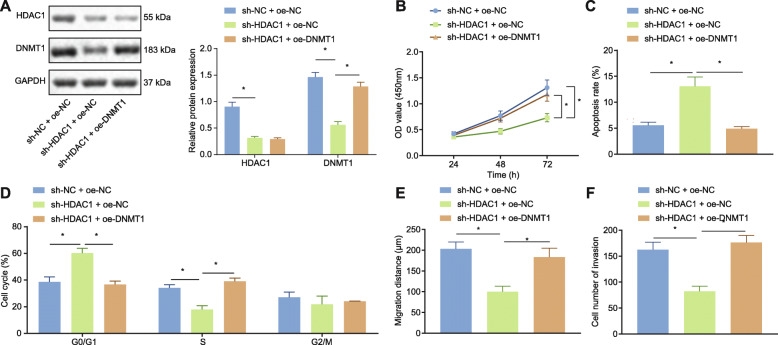
HDAC1 silencing downregulated the expression of DNMT1 to inhibit the proliferation of LAD cells. A549 cells were transfected with sh-NC + oe-NC, sh-HDAC1 + oe-NC or sh-HDAC1 + oe-DNMT1. **a** The expression of HDAC1 or DNMT1 by Western blot analysis, normalized to GAPDH. **b** CCK-8 test to detect cell viability. **c** Flow cytometry to detect cell apoptosis. **d** Flow cytometry to detect cell cycle distribution. **e** Scratch test to detect cell migration ability. **f** Transwell assay to detect cell invasion. All experiments were done in triplicate. * *p* < 0.05

DNMT1 has been previously reported to promote the development of LAD [17]. We subsequently set out to investigate the role of HDAC1 and DNMT1 in the proliferation and migration of LAD. We initially evaluated the effects associated with the overexpression and silencing of HDAC1 or DNMT1 (Fig. 5a). The CCK-8 experiment results (Fig. 5b), flow cytometry (Fig. 5c, d), cell scratch test (Fig. 5e) and Transwell assay (Fig. 5f) revealed reduced cell viability, increased apoptosis rate, increased number of G0/G1 phase cells, decreased S-phase-arrested cells, reduced cell migration and invasion, while the overexpression of DNMT1 reversed the impact of sh-HDAC1 on the above-mentioned cell functions in the cells with silenced HDAC1. The GLC-82 cells exhibited similar changing tendency as depicted in Supplementary Figure 2. In summary, silencing HDAC1 inhibits the proliferation, migration and invasion of LAD cells as well as blocking cell cycle from progressing into S phase by negatively regulating DNMT1.

### DNMT1 inhibited KLF4 expression level by enhancing its methylation

**Fig. 6:**
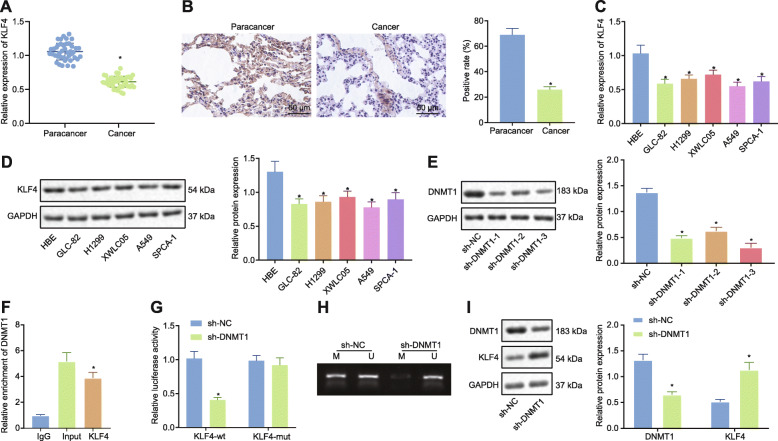
DNMT1 methylated KLF4 to inhibit its expression. **a** Expression of KLF4 in LAD tissues (*n* = 42) and paracancerous tissues (*n* = 42) by RT-qPCR, normalized to GAPDH. **b** Immunohistochemical detection of KLF4 in LAD tissues (*n* = 42) and paracancerous tissues (*n* = 42) (200 ×). **c** KLF4 mRNA expression in HBE cells and 5 LAD cell lines by RT-qPCR, normalized to GAPDH. **d** KLF4 protein expression in HBE cells and 5 LAD cell lines by Western blot analysis, normalized to GAPDH. **e** Silencing efficiency of DNMT1 by Western blot analysis, normalized to GAPDH. **f** ChIP test to detect the binding of DNMT1 to the KLF4 promoter region. **g** Dual luciferase reporter gene assay to detect the binding relation between DNMT1 and KLF4. **h** MSP to detect the effect of the methylation level of the KLF4 promoter region. **i** Western blot analysis of DNMT1 and KLF4 protein expression, normalized to GAPDH. * *p* < 0.05 vs. the paracancerous tissues (panels **a** and **b**), HBE cells (panels **c** and **d**), cells transfected with sh-NC (panels **e**, **i**), DNA-protein complex fragments incubated with IgG (panel **f**), or cells transfected with sh-NC and KLF4-WT (panel **g**). All experiments were done in triplicate

Previous reports have revealed that DNMT1 can inhibit KLF4 expression through methylating its promoter [12]. KLF4 has also been suggested to inhibit LAD [13]. We then set out to explore whether the inhibitory effect of KLF4 on LAD was regulated by DNMT1. Low levels of KLF4 expression were identified in the LAD tissues based on the RT-qPCR (Fig. 6a) and immunohistochemistry results (Fig. 6b). KLF4 expression at mRNA and protein level was significantly downregulated in five LAD cell lines relative to the HBE cell lines by RT-qPCR and Western blot analysis (Fig. 6c, d). Similarly, sh-DNMT1–3 exhibited higher silencing efficiency as per the Western blot analysis results (Fig. 6e). The ChIP assay (Fig. 6f) showed that DNMT1 was enriched on the KLF4 promoter. Dual luciferase reporter gene assay confirmed this result (Fig. 6g). In order to verify the methylation effect of DNMT1 on KLF4, MSP was employed to detect the methylation level of the KLF4 promoter region (Fig. 6h). The results demonstrated that the methylation level of KLF4 promoter region was significantly reduced after sh-DNMT1 treatment. Western blot analysis (Fig. 6i) revealed that KLF4 protein expression was significantly upregulated while DNMT1 protein level was reduced after sh-DNMT1 treatment. The above results provided evidence indicating that DNMT1 exerts its methylase function by binding to the KLF4 promoter region.

### DNMT1 silencing inhibited the proliferation, invasion, and migration of LAD cells by promoting KLF4 expression

**Fig. 7:**
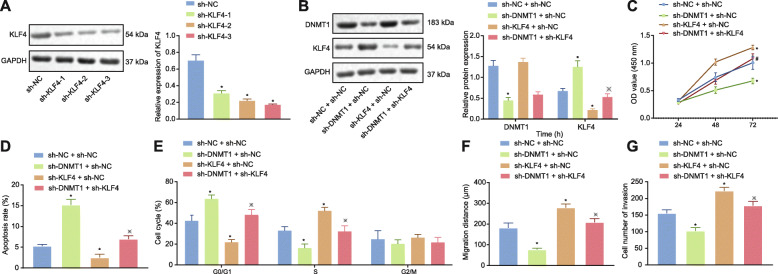
DNMT1-induced downregulation of KLF4 promoted the proliferation, migration, and invasion of LAD cells. **a** Western blot analysis to screen out the sequence with best silencing effects on KLF4 (normalized to GAPDH) following transfection with sh-NC, sh-KLF4–1, sh-KLF4–2, and sh-KLF4–3. A549 cells were transfected with sh-NC + sh-NC, sh-DNMT1 + sh-NC or sh-DNMT1 + sh-KLF4 for panels **b**-**g**. **b** The protein expression of DNMT1 or KLF4 by Western blot analysis, normalized to GAPDH. **c** CCK-8 test to detect cell viability. **d** Flow cytometry to detect cell apoptosis. **e** Flow cytometry to detect cell cycle distribution. **f** Scratch test to detect cell migration ability. **g** Transwell assay to detect cell invasion. All experiments were done in triplicate. * *p* < 0.05 vs. the sh-NC + sh-NC group (A549 cells transfected with sh-NC + sh-NC). # *p* < 0.05 vs. the sh-DNMT1 + sh-NC group (A549 cells transfected with sh-DNMT1 + sh-NC)

Next, we investigated the effect of DNMT1 on LAD by regulating KLF4. sh-KLF4–3 sequences with the higher silencing efficiency were screened out by Western blot analysis as the sequence for KLF4 silencing (Fig. 7a). Western blot analysis (Fig. 7b) provided further verification attesting that treatment with sh-DNMT1 led to a downregulation of DNMT1 and upregulation of KLF4 while sh-KLF4 treatment resulted in downregulation of KLF4 and no significant difference regarding DNMT1 expression. The results of CCK-8 experiment (Fig. 7c), flow cytometry (Fig. 7d, e), cell scratch test (Fig. 7f) and Transwell assay (Fig. 7g) indicated that silencing DNMT1 led to reduced cell viability, increased apoptosis rate, increased number of G0/G1 phase-arrested cells, decreased S-phase-arrested cells, reduced cell migration and invasion, while silencing KLF4 induced opposite results to those triggered by silencing DNMT1 as well as reversed the impact of sh-DNMT1 on the aforementioned cellular functions. Consistent results were observed in GLC-82 cells (Supplementary Figure 3). In summary, silencing DNMT1 inhibits the proliferation, migration and invasion of LAD cells in addition to inhibiting cell progression to the S phase through increasing the expression of KLF4.

### HOXA10 regulated DNMT1/KLF4 through HDAC1 to promote LAD in vivo

**Fig. 8:**
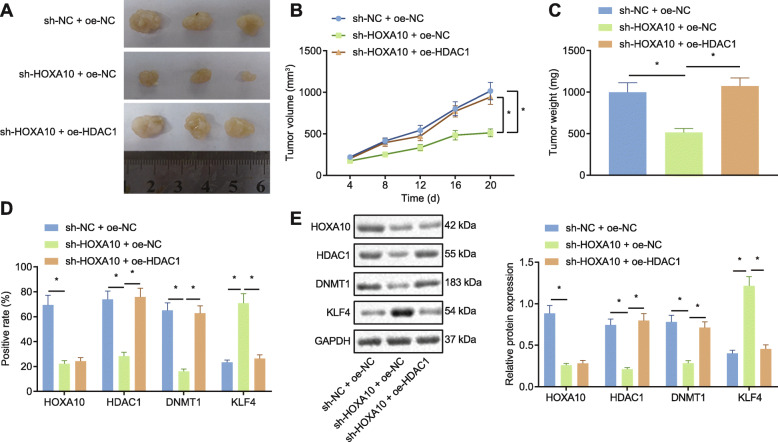
HOXA10-induced HDAC1 enhanced the tumorigenesis of LAD cells via DNMT1/KLF4. **a** The macroscopic appearance of the xenograft tumor implanted subcutaneously in mice. **b** The growth curve of the tumor volume in each group was graphed. **c** The tumor weight among the groups was compared. **d** Immunohistochemical detection of HOXA10, HDAC1, DNMT1, and KLF4 proteins in tumor tissue sections was performed (200 ×). **e** Western blot analysis of HOXA10, HDAC1, DNMT1, and KLF4 protein expression was performed (normalized to GAPDH). All experiments were done in triplicate. * *p* < 0.05

Finally, in vivo tumor xenograft experiments were performed with nude mice to elucidate the effects associated with HOXA10-mediated HDAC1 regulation of DNMT1/KLF4 on the tumorigenic ability of LAD. The transplanted tumors in nude mice are illustrated in Fig. 8a, with the tumor growth curve and tumor weight plotted on a graph (Fig. 8b, c). After silencing HOXA10, the results revealed that tumor growth was slowed while tumor weight was markedly diminished, while tumor growth was accelerated and the weight significantly elevated following HDAC1 overexpression. Immunohistochemistry (Fig. 8d) and Western blot analysis (Fig. 8e) findings demonstrated that after silencing HOXA10, HOXA10, HDAC1, and DNMT1 protein expression was significantly downregulated, while KLF4 protein expression was notably upregulated. However, following further overexpression of HDAC1, HDAC1 and DNMT1 expression was significantly upregulated, and KLF4 was significantly downregulated. The results illustrated that HOXA10 silencing inhibits the expression of DNMT1 by downregulating HDAC1, while stimulating the expression of KLF4, ultimately inhibiting the tumorigenic ability of LAD cells in vivo.

## Discussion

LAD represents the most common lung cancer subtype, and is often accompanied with a high rate of morbidity and mortality [18]. In spite of commendable advances in LAD therapy, patients are often diagnosed with this disease at an advanced or metastatic stage, resulting in a 5-year survival of less than 20% [19]. Thus, it is absolutely necessary to identify the genes and mechanisms related to LAD in order to improve diagnosis and treatment efficiency. In recent years, several articles have reported the regulation of HOXA10 in LAD [16, 20, 21]. Hence, the current study aimed to elucidate the downstream mechanism by which the HOXA10 influences and contributes to LAD. Our results demonstrated that HOXA10 promotes the malignant phenotypes of LAD via regulation of the histone deacetylase HDAC1-mediated DNMT1/KLF4 axis.

HOXA10 plays an important role in regulating cell differentiation, maturation, development and proliferation, and has been implicated in the occurrence and development of certain cancers [22–24]. A key initial finding of our study detected high levels of HOXA10 expression in LAD tissues and cell lines. We subsequently set out to investigate its effect on LAD phenotypes, and the results indicated that following HOXA10 silencing, cell viability, migration, and invasion were inhibited, cell cycle was arrested, and apoptosis increased. This finding was consistent with the report that HOXA10 overexpression may play an essential role in non-small cell lung carcinoma (NSCLC) tumorigenesis [20]. A previous study demonstrated that ELK1 induced upregulation of long noncoding RNA HOXA10-AS, which in turn activated the Wnt/β-catenin signaling and promoted LAD progression [21].

Next, the downstream mechanism of HOXA10 regulating lung cancer proliferation, migration, and invasion was further investigated. Our results illustrated that HOXA10 could directly bind to HDAC1 and subsequently promote its expression in LAD. Minamiya Y et al., emphasized higher HDAC1 expression as an independent marker of poor prognosis in patients with LAD [25]. In addition, HOXA10 silencing suppresses the proliferation of hepatoma cells, induces cell cycle arrest and apoptosis by upregulating HDAC1 transcription [10], which was consistent with our findings.

Johann C Brandes et al. concluded that class I HDACs are mediators that stabilize DNMT1 and are promising targets for the prevention of lung cancer induced by smoke carcinogens [26]. Subsequent results in our study revealed that the expression of DNMT1 was higher in tumor samples, highlighting the correlation between DNMT1 and LAD. Consistent with our observations, a previous study indicated that DNMT1 was overexpressed in primary NSCLCs [27]. We then speculated that HDAC1 could potentially promote the proliferation, migration and invasion of LAD via DNMT1, which we subsequently verified through a HDAC1 silencing experiment as well as a DNMT1 rescue experiment. We then explored the interaction between HDAC1 and DNMT1 and our data indicated that HDAC1 inhibited the degradation of DNMT1 by strengthening its deacetylation, a finding of which was consistent with existing literature that suggests that DNMT1 was stabilized by HDAC1 [11].

Bearing in mind the function of DNMT1 as a DNA methyltransferase, we subsequently set out to identify the catalytic substrate of DNMT1. Previous research has suggested that upregulation of DNMT1 and promoter hypermethylation may triggered a downregulation in the expression of KLF4 [28]. Additionally, KLF4 is found to act as important player in the progression of many aggressive cancers, such as lung cancer [29, 30]. Our results provided further evidence attesting the notion that KLF4 was indeed associated with LAD, with poorly expressed KLF4 identified in LAD. Functionally, we also observed that by silencing DNMT1, KLF4 methylation was increased as reflected by activation of its expression. Moreover, downregulation of DNMT1 could suppress cancer cell viability, migration and invasion through upregulation of KLF4 expression. Our observation was similar to the description that DNMT1 might reduce KLF4 expression by catalyzing DNA methylation in the promoter region of KLF4 [31]. Furthermore, DNMT1 inhibition reduces KLF4 promoter DNA methylation and activates KLF4 expression in pancreatic cancer cells [28].

## Conclusion

**Fig. 9:**
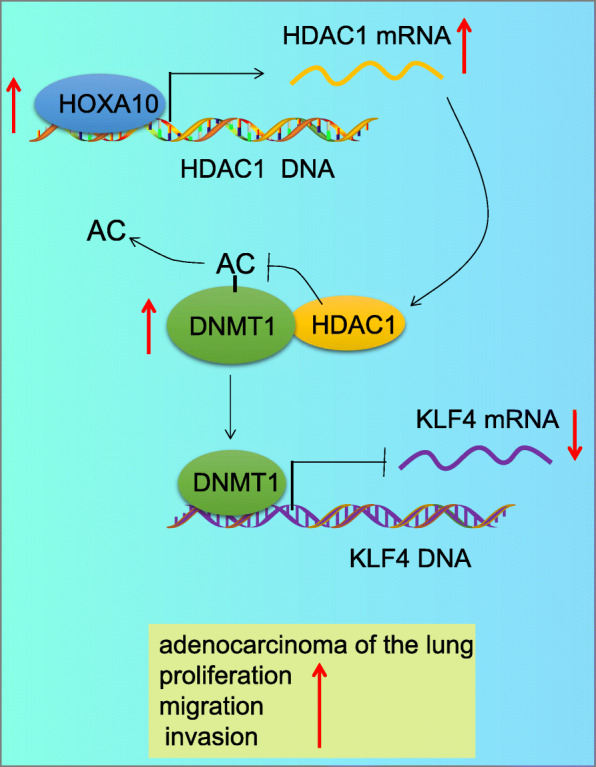
The graphical summary of the function and mechanism of HOXA10 in LAD that silencing HOXA10 exerts inhibitory effect on the occurrence of LAD by reducing KLF4 methylation through promoting on DNMT1 degradation via downregulation of HDAC1

Altogether, the central observations of our study provide evidence highlighting the strong association between HOXA10 expression and LAD progression in vitro and in vivo (Fig. 9). Our results demonstrate that HOXA10 knockdown downregulated HDAC1 transcription, which enhanced DNMT1 acetylation, thus reducing KLF4 methylation. HDAC1 mediated the effects of HOXA10 on cancer cell proliferation, migration, invasion, cell cycle progression, and apoptosis. Our findings provide a fresh perspective into the mechanisms underlying the pathogenesis of LAD as well as potential therapeutic targets for the disease. Nevertheless, due to limited tumor material, variation of degree of differentiation and clinical stage, the representativeness of the samples may be insufficient. Hence, further study into HOXA10 and HDAC1 expression in progression and prognosis of LAD warrants further study. Also, future investigations are needed to further explore new ideas on the mechanism by which HOXA10 contributes to the development of LAD.

## Supplementary Information

**Additional file 1: Supplementary Figure 1.** HDAC1 is the mediator of HOXA10 in GLC-82 cells to regulate proliferation, apoptosis, invasion and migration. GLC-82 cells were transfected with sh-NC + oe-NC, sh-HOXA10 + oe-NC or sh-HOXA10 + oe-HDAC1. A, HOXA10 and HDAC1 protein expression by Western blot analysis, normalized to GAPDH. B, Cell viability detection by CCK-8 test. C, The detection of cell apoptosis by flow cytometry. D, The cell cycle distribution by flow cytometry. E, Cell migration by scratch test (40 ×). F, Cell invasion by Transwell assay (200 ×). All experiments were done in triplicate. * *p* < 0.05.

**Additional file 2: Supplementary Figure 2.** HDAC1 silencing downregulated the expression of DNMT1 to inhibit the proliferation of GLC-82 cells. GLC-82 cells were transfected with sh-NC + oe-NC, sh-HDAC1 + oe-NC or sh-HDAC1 + oe-DNMT1. A, The expression of HDAC1 or DNMT1 by Western blot analysis, normalized to GAPDH. B, CCK-8 test to detect cell viability. C, Flow cytometry to detect cell apoptosis. D, Flow cytometry to detect cell cycle distribution. E, Scratch test to detect cell migration ability. F, Transwell assay to detect cell invasion. All experiments were done in triplicate. * *p* < 0.05.

**Additional file 3: Supplementary Figure 3.** DNMT1-induced downregulation of KLF4 promoted the proliferation, migration, and invasion of GLC-82 cells. GLC-82 cells were transfected with sh-NC + sh-NC, sh-DNMT1 + sh-NC or sh-DNMT1 + sh-KLF4. A, The protein expression of DNMT1 or KLF4 by Western blot analysis, normalized to GAPDH. C, CCK-8 test to detect cell viability. D, Flow cytometry to detect cell apoptosis. E, Flow cytometry to detect cell cycle distribution. F, Scratch test to detect cell migration ability. G, Transwell assay to detect cell invasion. All experiments were done in triplicate. * *p* < 0.05.

## Data Availability

All data generated or analyzed during this study are included in this published article and its supplementary information files.

## Abbreviations

LAD: Lung adenocarcinoma
HOXA10: Homeobox A10
KLF4: Kruppel-like factor 4
HDAC1: Histone deacetylase 1
DNMT1: DNA methyltransferase 1
FBS: Fetal bovine serum
NC: Negative control
shRNAs: Short hairpin RNAs
RT-qPCR: Reverse transcription quantitative polymerase chain reaction
GAPDH: Glyceraldehyde-3-phosphate dehydrogenase
SDS-PAGE: Sodium dodecyl sulfate-polyacrylamide gel electrophoresis
CCK-8: Cell counting kit-8
FITC: Fluorescein isothiocyanate
7-ADD: 7-AminoactinomycinD
PI: Propidium iodide
WT: Wild-type
MUT: Mutant
Ub: Ubiquitination
ChIP: Chromatin immunoprecipitation
Co-IP: Co-immunoprecipitation
MSP: Methylation-specific PCR
ANOVA: Analysis of variance
NSCLC: Non-small cell lung carcinoma
